# The Treatment of Snake Bites in a First Aid Setting: A Systematic Review

**DOI:** 10.1371/journal.pntd.0005079

**Published:** 2016-10-17

**Authors:** Bert Avau, Vere Borra, Philippe Vandekerckhove, Emmy De Buck

**Affiliations:** 1 Centre for Evidence-Based Practice (CEBaP), Belgian Red Cross-Flanders, Mechelen, Belgium; 2 Department of Public Health and Primary Care, Faculty of Medicine, KU Leuven, Leuven, Belgium; 3 Faculty of Medicine and Health Sciences, Ghent University, Ghent, Belgium; Institut de Recherche pour le Développement, BENIN

## Abstract

**Background:**

The worldwide burden of snakebite is high, especially in remote regions with lesser accessibility to professional healthcare. Therefore, adequate first aid for snakebite is of the utmost importance. A wide range of different first aid techniques have been described in literature, and are being used in practice. This systematic review aimed to summarize the best available evidence concerning effective and feasible first aid techniques for snakebite.

**Methods:**

A systematic literature screening, performed independently by two authors in the Cochrane Library, MEDLINE and Embase resulted in 14 studies, fulfilling our predefined selection criteria, concerning first aid techniques for snakebite management. Data was extracted and the body of evidence was appraised according to the GRADE approach.

**Principal findings:**

The pressure immobilization technique was identified as the only evidence-based first aid technique with effectiveness on venom spread. However, additional studies suggest that proper application of this technique is not feasible for laypeople. Evidence concerning other first aid measures, such as the application of a tourniquet, suggests avoiding the use of these techniques.

**Conclusions:**

The practical recommendation for the treatment of snakebite in a first aid setting is to immobilize the victim, while awaiting the emergency services. However, given the low to very low quality of the data collected, high quality randomized controlled trials concerning the efficacy and feasibility of different variations of the pressure immobilization technique are warranted.

## Author Summary

The Belgian Red Cross-Flanders develops first aid guidelines that specifically target laypeople. In the context of updating the first aid guidelines for sub-Saharan Africa, we aimed to summarize the best available evidence for the treatment of snakebite, feasible for laypeople. Of the numerous first aid measures supported in literature and used in practice, we could only find evidence concerning effectiveness for the pressure immobilization technique on the spread of snake venom, which involves application of a firm pressure bandage on the bitten limb, together with immobilization of the limb. However, studies concerning its feasibility suggest this technique is difficult for laypeople to apply correctly. Keeping the limb immobilized on the other hand had a beneficial effect on the spread of the venom. However, given the low to very low quality of the evidence, high quality trials concerning the effectiveness and feasibility of different variations of the pressure immobilization technique are needed.

## Introduction

Venomous snakes occur worldwide, with the exception of a few remote islands, regions of high altitude and the arctic regions [1]. Not surprisingly, ophidiophobia, or fear of snakes, is commonly reported [2]. It has also been demonstrated that humans are able to detect snakes faster than other, less harmful stimuli, suggesting the presence of an internal, evolutionary conserved warning system [3,4]. Despite this, snakebites occur frequently, with a global estimate of 421,000 to 1,842,000 cases of snake envenomation and 20,000 to 94,000 deaths each year [5]. The prevalence is especially high in the tropical regions of South and Southeast Asia, Latin America and sub-Saharan Africa, with estimates of 13.33, 12.59 and 11.11 cases of snakebite/100,000 inhabitants, respectively. However, the accuracy of these numbers has been questioned [6,7]. Furthermore, studies in which data was collected through household surveys instead of official records suggested that the actual incidence of snakebite might be even higher, as many snake bitten subjects fail to present themselves to healthcare centers due to remoteness or a preference for traditional healers [7]. Snakebite victims that survive their encounter with a snake often also suffer from permanent disability. Several snake venoms, such as those from vipers and some cobra species, induce local necrosis, which can lead to amputations [8], further increasing the estimated burden of snakebite [9].

Different studies have shown that people living in rural areas are at higher risk of encountering snakebite than people living in urban areas [7,10,11]. This might be due to a higher presence of snakes in rural areas, but also due to occupational hazards, as many people living in rural areas are occupied in agriculture, which has been shown to be a risk factor for snakebite [10–13]. Furthermore, snakebite victims are often adult males in the professionally active age range [10–12]. Therefore, snakebite is considered to be a condition with a high economic impact in an economically vulnerable population [14].

The high burden of snakebite and the fact that snakebite mostly occurs in rural areas, with less accessibility to professional health care and therefore rapid antivenom therapy, illustrate that adequate first aid treatments are of the utmost importance for achieving a positive outcome on both mortality and morbidity after a snakebite. In literature, many different techniques, and a combination thereof, are claimed to be effective for the treatment of snakebite [15,16]. These include techniques suggested to deactivate the venom, such as the application of electroshocks, cryotherapy or the use of traditional medicine and concoctions, a collection of practices where mixtures of herbs, oils and other products are being ingested or applied to the bite wound. Furthermore, techniques which are supposed to remove venom from the bite wound include suction of the wound, by mouth or specialized suction devices, incision/excision of the bite wound, irrigation of the bite wound, or the use of “snake stones”, which are believed to absorb the poison out of the wound. Methods proposed to limit the spread of the venom in the body include application of a tourniquet, which completely blocks the blood flow to the bitten limb, and the pressure immobilization technique. The latter technique involves application of a pressure bandage at sufficiently high pressures to block lymphatic flow, but without actually applying a tourniquet, together with immobilization of the bitten limb [17]. This systematic review is the first in its kind to synthesize the available evidence concerning suggested first aid measures for snakebite, thus facilitating evidence-based decision making during the development of snakebite first aid guidelines for laypeople. For this, the following PICO question was formulated: In people with snakebites (P), is a certain first aid intervention (I), compared to another first aid intervention or no intervention (C), effective and feasible for laypeople as a first aid treatment to increase survival, tissue healing, functional recovery, pain, complications, time to resumption of usual activity, restoration to the pre-exposure condition, time to resolution of the symptoms or other health outcome measures (including adverse effects) (O)?

## Methods

We reported our systematic review according to the reporting criteria provided in the PRISMA checklist (S1 Table) [18]. No protocol was filed prior to the preparation of the manuscript, however the methodology described in our previously published methodological charter was followed [19].

### Search strategy developed to identify studies relevant to the PICO question

The following databases were searched for relevant studies from their date of inception to March 10, 2016: The Cochrane Library for clinical trials and systematic reviews, MEDLINE (using the PubMed interface) for systematic reviews, experimental and observational studies and Embase (via the Embase.com interface) for systematic reviews, experimental and observational studies, using the search strategies described in S1 File. Titles and abstracts of retrieved articles were scanned, and for relevant articles the full-texts were obtained and studied. Studies that did not meet the predefined selection criteria, as described below, were excluded. The reference lists of included studies and also the first 20 similar articles in PubMed were screened for other relevant publications. The searches and study selection procedures were performed independently by two reviewers (BA and VB). Any discrepancy between the reviewers was resolved by consensus or by consulting a third reviewer (EDB).

### Predefined criteria used to select studies addressing the PICO question

For the population (P), studies concerning people with snakebites or healthy volunteers with “mock” snakebites were included. The interventions (I) that were included in this systematic review were interventions for the first aid management of snakebites that can be applied by laypeople without medical background. We excluded interventions for the management of snakebites that are not feasible to be performed in a first aid setting where laypeople are the first aid providers. We selected studies that compared (C) the interventions to any other first aid intervention or no intervention. Concerning the outcomes (O), we included (1) survival, functional recovery, pain, complications, time to resumption of usual activity, restoration of the pre-exposure condition, time to resolution of symptoms or other health outcome measures (including adverse effects) for studies involving snakebite victims, (2) spread of mock venom for studies investigating the efficacy of pressure immobilization and (3) quality of the bandage applied and tension generated for studies investigating the feasibility of pressure immobilization.

The following experimental study designs were included: (quasi or non-) randomized controlled trials, controlled before and after studies or controlled interrupted time series, if the data were available. For studies concerning the feasibility of first aid interventions, non-controlled before and after studies were also included, since this is typically measured with that type of study design. Observational studies of the following types were also included: cohort and case-control study, controlled before and after study or controlled interrupted time series, if the data were available. We excluded observational studies if the intervention was already studied in experimental studies, letters, comments, narrative reviews, case reports, cross-sectional studies, animal studies, ex vivo or in vitro studies, conference abstracts unless no other relevant data was available, studies reporting no quantitative data, studies reporting only means, but no standard deviations (SDs), effect sizes, p-values. Only studies reported in English were selected.

### Data collection from studies meeting the selection criteria

Data concerning study design, study population, outcome measures (expressed as mean difference, odds ratio or risk ratio) and study quality were independently extracted from the included studies by two reviewers (BA and VB) using an in advance prepared form. Any discrepancy between the reviewers was resolved by consensus. Data and p-values were extracted directly from the publications, unless it is stated that these were calculated from raw data available using the Review Manager software [20]. Outcomes from the selected studies without raw data or statement of significance were not extracted. Data are represented as mean±standard deviation (SD) or relative risk (RR) with 95% CI (confidence interval), unless otherwise stated.

### Quality assessment of the evidence using the GRADE approach

The overall quality of “the body of evidence” was determined using the GRADE approach [21]. Evidence from experimental studies started with an initial “high” quality level, and evidence from observational studies with an initial “low” quality level. The evidence was then assessed for limitations in 5 domains, for which the quality of evidence could be downgraded, namely limitations in study design, indirectness, imprecision, inconsistency and reporting bias. Limitations in study design were assessed at the level of the individual study using the items listed by GRADE. The overall quality was assessed separately for (1) experimental studies concerning efficacy of pressure immobilization, (2) experimental studies concerning feasibility of the application of pressure immobilization to be performed by laypeople and (3) observational studies concerning other first aid techniques (tourniquet application, suction, traditional medicine, snake stones, incision of the bite wound).

## Results

### Process of study selection

**Fig 1:**
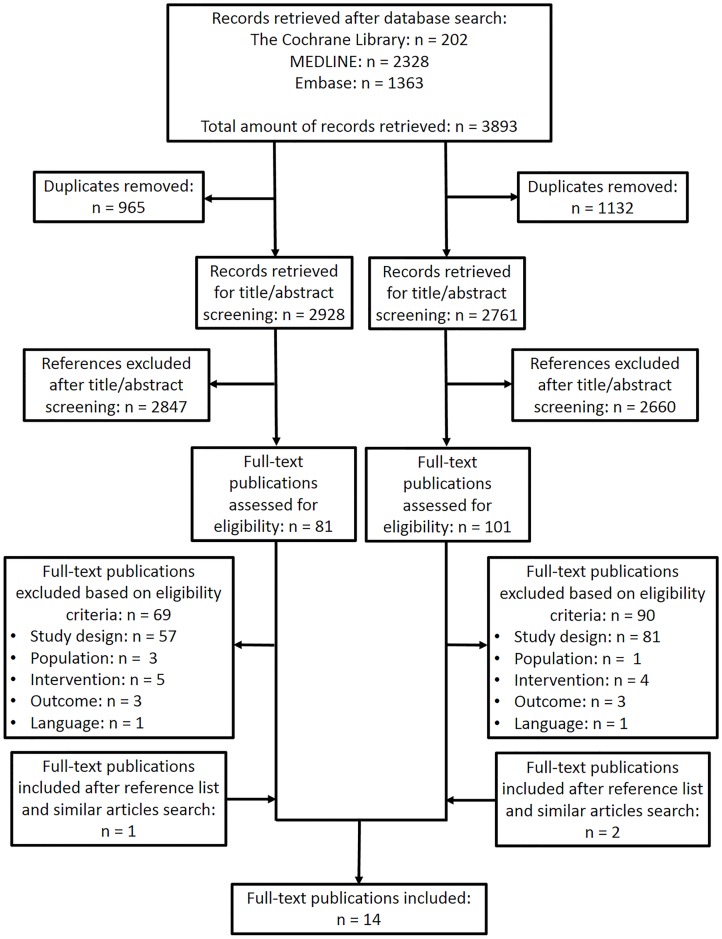
PRISMA flowchart for the selection of eligible studies.

A search in The Cochrane Library, MEDLINE and Embase resulted in a total of 3,893 retrieved references (Fig 1). After removing 956 (BA) and 1,132 (VB) duplicates, the titles and abstracts of 2,928 (BA) and 2,761 (VB) records were screened on relevance regarding the PICO question. For 81 (BA) and 101 (VB) publications, a full-text was obtained and eligibility was assessed, resulting in 12 articles that matched the predefined selection criteria. The majority of publications excluded had an inappropriate study design. A search in the references and similar articles lists of these publications resulted in 2 additional publications matching the selection criteria, leading to a total of 14 included articles. An overview of the in- and excluded studies can be found in Table 1 and S2 Table, respectively.

**Table 1:** Characteristics of the included studies.

Author, year, Country	Study design	Population	Comparison	Remarks
Amaral, 1998, Brazil [22]	Observational: Retrospective cohort study	97 patients who presented to Hospital João XXIII in Belo Horizonte between January 1991 and December 1996 after being bitten by the South American rattlesnake. 16 females, 81 males, age range 3–88 years. Patients were divided in a tourniquet group (n = 45, mean age 35.5±19.6 years) and a non-tourniquet group (n = 52, mean age 28.4±179 years)	**Intervention:** Tourniquet versus **Control:** no tourniquet	
Anker, 1982, Australia [23]	Experimental: Non-randomized controlled trial	12 healthy volunteers (n = 3 per group), aged 19–31 years, were subcutaneously injected in their leg with a radioactive “mock” venom, consisting of saline, containing 0.2 or 0.3 μCi/kg Na^131^I	**Interventions:** 1. “CSL (Commonwealth Serum Laboratories) method”: treatment for +/-60 min with crepe or elastic bandage (pressure 55±5 mmHg) and a padded straight wooden splint to the medial side of the lower limb (pressure immobilization); 2. Pneumatic splint: treatment for +/-60 min with a full-length lower limb air splint, pressure maintained at 55 mmHg; 3. Treatment for +/-60 min with a pressure cloth over the injection site (>70 mmHg pressure). The pad was held in place by 2 broad bandages firmly binding it to the leg (“Monash method”) versus **Control:** No treatment [Data of pneumatic splint were not extracted]	
Anker, 1983, Australia [24]	Experimental: Non-randomized controlled trial	12 healthy volunteers (n = 3 per group), aged 18–28 years, were subcutaneously injected in their leg with a radioactive “mock” venom, consisting of saline, containing 0.2 μCi/kg ^125^I-labeled porcine insulin	**Interventions:** 1. “CSL method”: treatment for +/-60 min with crepe or elastic bandage (pressure 55±5 mmHg) and a padded straight wooden splint to the medial side of the lower limb (pressure immobilization); 2. Pneumatic splint: treatment for +/-60 min with a full-length lower limb air splint, pressure maintained at 55 mmHg; 3. Treatment for +/-60 min with a pressure cloth over the injection site (>70 mmHg pressure). The pad was held in place by 2 broad bandages firmly binding it to the leg (“Monash method”) versus **Control:** No treatment [Data of pneumatic splint were not extracted]	
Bhat, 1974, India [30]	Observational: Prospective cohort study	310 viper bitten subjects presenting at the S.M.G.S Hospital in Jammu in the period 1978–1981, (271 men and 39 women), 6% < 10 years; 80% between 10 and 55 years and 14% above 55 years	**Interventions:** 1. Tourniquet (n = 78); 2. Tourniquet with incision (n = 71); 3. Incision (n = 13) versus **Control:** No first aid (n = 148)	
Canale, 2009, Australia [27]	Experimental: Non-randomized controlled trial	96 participants, 78 health care workers and 18 laypeople, were asked to apply a pressure bandage to a human lower limb in a simulated setting of a snakebite	**Intervention:** Training (n = 36) versus **Control:** no training (n = 36) in applying elasticized bandage	
França, 2003, Brazil [31]	Observational: Prospective cohort study	137 lance-headed viper bitten subjects, presenting at the Hospital Vital Brazil in São Paulo between 1989 and 1990 (101 men and 36 women, mean age 30.12±16.58 years), with ‘mild’ local envenomation (swelling in 1–2 segments of the bitten limb with or without coagulopathy) or ‘moderate’ local envenomation (swelling in 3–4 segments of the bitten limb with or without coagulopathy)	**Intervention:** Tourniquet (n = 56) versus **Control:** no tourniquet (n = 61)	
Howarth, 1994, Australia [25]	Experimental: Controlled trial (within subjects design with a non-treated comparison group)	24 participants, of which 15 healthy volunteers (9 men and 6 women, aged 24–47 years) and 9 people undergoing lymphoscintigraphy, but with confirmed normal lymphatic circulation (2 men and 7 women, aged 24–70 years). The healthy volunteers received subcutaneous injections in forearms and lower legs, the people undergoing lymphoscintigraphy intradermal injections in hands and feet of 0.1 ml 10 MBq ^99m^technetium antimony sulfur colloid	A. **Intervention:** Crepe bandage (pressure 50–70 mmHg) and splint; on one lower and one upper limb versus **Control**: unbandaged limb. B. **Intervention:** Crepe bandage (pressure 50–70 mmHg) and splint on upper or lower limb in rest versus **Control:** while walking [Only data from in rest versus while walking was extracted]	
Khin Ohn Lwin, 1984, Myanmar [32]	Observational: Prospective cohort study	38 Russell’s viper bitten subjects, presenting at the Insein Hospital in Yangon between 30 October 1981 and 21 July 1982, male:female ratio 5:1, aged 28–55 years	**Intervention:** Tourniquet (n = 20) versus **Control:** no tourniquet (n = 18)	Serum venom levels were measured at time intervals before and after antivenom treatment in patients with and without tourniquet placed. [Only data from before antivenom treatment were extracted]
Madaki, 2005, Nigeria [13]	Observational: Retrospective cohort study	103 snakebite patients, 62 males, 41 females, mean age 26.8±14.8 years at Zamko Comprehensive Health Care Centre between January and December 2001	**Interventions:** 1. Tourniquet (n = 35); 2. Ingested or applied traditional medicine (n = 40); 3. Snake stone (n = 11) versus **Control:** No first aid (n = 19)	The n-value for group 2: ingested or applied traditional medicine was reported in the text, but differs from the n-value reported in the table with the distribution of first aid use. Therefore it is unclear whether this value is correct
Michael, 2011, Nigeria [16]	Observational: Prospective cohort study	72 snakebite patients, 52 males, 20 females; mean age 23.4±15.7 years at Zamko Comprehensive Health Care Centre between April and July 2006	A. Different first aid techniques applied: **Interventions:** 1. Tourniquet (n = 53); 2. Applied concoction (n = 15); 3. Ingested concoction (n = 10); 4. Incision (n = 8); 5. Snake stone (n = 4); 6. Suction (n = 3) versus **Control:** No first aid (n = 15). B. Timing of presentation: **Intervention:** Early (<4h) (n = 28) versus **Control:** late (>4h) (n = 44) presentation after the bite [Data from early vs late presentation was not extracted]	A minimum sample size of 60 subjects was targeted, based on 80% power to detect a difference in complication rate with 95% confidence
Norris, 2005, USA [28]	Experimental: Non-randomized trial	40 participants (20 emergency medicine physicians and 20 laypeople) each performed pressure immobilization with an elastic bandage 5 times (own non-dominant arm, own dominant arm, own dominant leg, arm investigator, leg investigator). Lay volunteer applications (n = 100) were compared with medical volunteer application (n = 100)	Ability to apply correctly the pressure immobilization technique was compared between **Intervention:** healthcare workers and **Control:** laypeople	Pressure was measured by skin interface pressure (SIP) measurement device placed at the simulated snakebite
Simpson, 2008, India [29]	Experimental: Randomized controlled trial	40 volunteers (32 males and 8 females) were randomized into 2 groups. Group 1 (n = 20), 15 males and 5 females, mean age 44±15.6 years, received only written instructions; group 2 (n = 20), 17 males and 3 females, mean age 41.5±11.1 years received further specific training	Ability to correctly apply the pressure immobilization technique was compared between **Intervention:** people that received an intense training (“cone of learning” model) and **Control:** people that received the written instructions	
Tun Pe, 1994, Myanmar [26]	Experimental: Non-randomized controlled trial	22 healthy, male volunteers, mean age 35 years (range 22–58 years), receiving subcutaneously injected radioactive labeled “mock venom” in the lower limb, consisting of 12 or 20 μCi Na^131^I	**Intervention:** Pad-treated group (n = 14): firm rubber pressure pad (variant of the Monash method) and cotton bandage was applied immediately over the site of injection. The limb was immobilized with bamboo splints versus **Control:** No treatment (n = 8) Duration of treatment: 45–79 min	
Wang, 2014, China [33]	Observational: Retrospective cohort study	292 Chinese cobra bite patients, presenting at the First Affiliated Hospital of the Guangxi Medical University in Nanning, 257 males and 42 females, mean age 39.7±11.5 years	**Intervention:** Tourniquet (n = 220) versus **Control:** no tourniquet (n = 72)	

### Characteristics of the included studies

Of the 14 included articles, 7 were experimental [23–29] and 7 were observational studies [13,16,22,30–33]. 4 experimental studies evaluated the efficacy of a first aid treatment, i.e. variants of the pressure immobilization technique, on simulated snake bites [23–26], while 3 others examined the feasibility of pressure immobilization to be performed by laypeople [27–29]. The observational studies all examined the outcomes of different applied first aid procedures in snakebite patients [13,16,22,30–33]. An overview of the study characteristics of the included studies is shown in Table 1.

Two controlled trials performed by Anker *et al*. examined the effectiveness of two different pressure immobilization techniques on the spread of a subcutaneously injected radioactive “mock venom”, consisting of either saline, containing 0.2 or 0.3 μCi/kg Na^131^I [23], or saline, containing 0.2 μCi/kg ^125^I-labeled porcine insulin [24], in the leg of healthy volunteers. Both studies compared the effectiveness of a crepe or elastic compression bandage (55±5 mmHg) and a wooden splint on the injected limb or a cloth pad bound firmly over the site of injection (>70 mmHg) with 2 broad bandages to no first aid treatment.

A study by Tun Pe *et al*. used a variant of the pressure cloth studied by Anker *et al*., namely a rubber pad instead of a cloth pad bound firmly at the site of injection, together with immobilization of the injected limb by splinting [26]. The effectiveness of this first aid technique was assessed on the spread of a mock venom, consisting of 12 or 20 μCi Na^131^I, injected subcutaneously in the leg of healthy volunteers and compared to no first aid treatment.

Howarth *et al*. used a within subjects design to study the effect of pressure immobilization, a crepe bandage of 50–70 mmHg with a splint applied to one upper and one lower limb, on the spread of a mock venom, consisting of 0.1 ml 10 MBq ^99m^technetium antimony sulfur colloid, subcutaneously injected in both upper and lower limbs of healthy volunteers [25]. The spread of the mock venom was compared between the treated limbs and the corresponding untreated limbs, both in rest and during walking.

Norris *et al*. compared the correct application of a pressure bandage on the own or the investigator’s upper and lower limbs between a group of health care workers and a group of laypeople after receiving only written instructions [28]. In contrast, Canale *et al*. studied whether intense training influenced the correct application of an elastic bandage on a simulated snakebite victim’s lower limb by a test group consisting of both healthcare workers and laypeople, compared to before training, when no instructions were given [27]. Simpson *et al*. performed a randomized controlled trial comparing the correct application of an elastic bandage on the upper or lower limb of a simulated snakebite victim by two groups of volunteers receiving either intense training or only written instructions [29]. Furthermore, the retention of the acquired skills was measured in the group receiving intense training immediately versus three days after receiving the training.

The observational studies included are cohort studies describing outcomes following the application of different first aid techniques in snakebite victims presenting at health care facilities after being bitten by South American rattle snakes [22], vipers [30], lance-headed vipers [31], Russell’s vipers [32], Chinese cobras [33] or unspecified snakes [13,16]. The use of a tourniquet was studied in all 7 observational studies, while 2 studies also looked at the effects of incision [16,30], 2 studies examined the use of “snake stones” and traditional medicine and concoctions [13,16] and one study examined the use of suction [16].

### Synthesis of findings from the included studies

A structured synthesis of the findings from the included studies can be found in S3 Table, a narrative overview is given below.

#### Pressure Immobilization

The experimental studies by Anker *et al*. [23] and Tun Pe *et al*. [26] used the time to reach 80% of the maximal radioactivity in the blood after mock venom injection as outcome for comparing different types of pressure immobilization techniques. If no first aid technique was applied, this amount was reached after 26±3.61 min in the study of Anker *et al*. [23]. Pressure immobilization using an elastic bandage with a splint was not shown to be effective, as the time to reach 80% of the maximal radioactivity in the blood was 26±17.06 min (mean difference (MD) = 0.0, 95%CI [-19.73;19.73], p = 1). In contrast, using a firmly bound cloth pad over the site of injection delayed the time to reach 80% of the maximal radioactivity in the blood to 74.3±3.79 min (MD = 48.3, 95%CI [42.38;54.22], p<0.001). Similarly, Tun Pe *et al*. found that the use of a firmly bound rubber pad over the site of injection, together with splinting resulted in a delay of the time to reach 80% of the maximal radioactivity in the blood from 42.38±5.01 min (no treatment) to 66.07±9.71 min (MD = 23.69, 95%CI [17.53;29.85], p<0.001) [26]. The second study by Anker *et al*., in which a more “physiological” mock venom was used, compared the amount of radioactivity in the blood after 60 min, as % of the maximal radioactivity measured [24]. Corresponding to the previously mentioned studies, it was not shown that an elastic bandage with splinting was effective to decrease the amount of radioactivity in the blood (40.67±4.51%), compared to no treatment (46.33±16.17%, MD = -5.66, 95%CI [-24.66;13.34], p = 0.56), while a firmly bound cloth pad was found to be effective (4.67±3.25%, MD = -41.66, 95%CI [-60.32;-23.0], p<0.0001). In the study by Howarth *et al*., studying pressure immobilization with an elastic bandage and splinting, it was shown that rest resulted in a significant decrease in the proportion of volunteers with tracer transit compared to the proportion of volunteers with tracer transit while walking in both lower limbs (4/13 vs 9/9, RR: 0.34, 95%CI [0.16;0.73], p = 0.006) and upper limbs (7/13 vs 6/6, RR: 0.58, 95%CI [0.34;0.98], p = 0.04) [25].

Norris *et al*. were the first to investigate the feasibility of the application of pressure immobilization by comparing the proportions of correct pressures generated and correct applications of an elastic bandage between laypeople and healthcare workers [28]. No difference was found concerning the correct applications (5/100 vs 13/100, RR:0.38, 95%CI [0.14;1.04], p = 0.06) nor the correct pressures generated (14/100 vs 17/100, RR: 0.82, 95%CI [0.43; 1.58], p = 0.56). In the study by Canale *et al*., it was shown that training had a beneficial influence on the proportion of tensions generated within the correct range using an elastic bandage (18/36), compared to no training (5/36, odds ratio (OR): 6.20, p = 0.002) [27]. A similar observation has been made by Simpson *et al*., who noticed that the proportion of tensions generated within the correct range using an elastic bandage was significantly higher in a test group of volunteers receiving intense training (12/20), compared to a control group receiving only written instructions (0/20, RR: 25.0, 95%CI [1.58;395.48], p = 0.02) [29]. Accordingly, the pressures generated by the test group were significantly higher (57.7±17.0 mmHg) than those generated by the control group (10.5±11.0 mmHg, MD = 47.2, 95%CI [38.33;56.07], p<0.001). Finally, this study also demonstrated the lack of retention of the ability to correctly apply the elastic bandage, as it was shown that the proportion of tensions generated within the correct range, 3 days after the training (25%), was significantly lower than 1 h after the training (60%, 95%CI [6%;44%], p<0.001).

#### Tourniquet

The study by Bhat found a significantly increased incidence of local swelling in snakebite victims treated with a tourniquet (78/78, RR: 1.71, 95%CI [1.49;1.96], p<0.001) or a tourniquet with incisions in the wound (71/71, RR: 1.71, 95%CI [1.49;1.96], p<0.001), compared to snakebite victims receiving no first aid (86/148) [30]. França *et al*. described a significantly increased odds for an increased severity of local envenomation in snakebite victims receiving a tourniquet, compared to those not receiving a tourniquet (adjusted OR: 4.31, 95%CI, [1.33;13.89], p = 0.015) [31]. Furthermore, Wang *et al*. showed a significantly increased risk of skin grafting needed in snakebite victims treated with a tourniquet (44/220), compared to those not treated with a tourniquet (7/72, RR: 2.06, p = 0.046) [33].

No significant differences were found between snakebite victims treated with a tourniquet (with or without additional incisions in the bite wound) and victims who received no tourniquet or no first aid for the following outcomes: acute renal failure (4/42 vs 4/52, RR: 1.24, 95%CI [0.33;4.66], p = 0.75) [22], acute respiratory failure (3/35 vs 3/49, RR: 1.4, 95%CI [0.3;6.53], p = 0.67) [22], death (2/45 vs 3/52, RR: 0.77, 95%CI [0.13;4.41], p = 0.77) [22], local edema (17/42 vs 21/51, RR: 0.98, 95%CI [0.6;1.61], p = 0.95) [22], occurrence of hemorrhagic syndrome (49/78 vs 98/148, RR: 0.95, 95%CI, [0.77;1.17], p = 0.62) [30], concentration of venom in the serum (77.85±74.82 ng/mL vs 60.88±39.39 ng/mL, MD = 16.97, 95%CI [-20.79;54.73], p = 0.38) [32] (197.7±230.4 ng/mL vs 283.5±406.6 ng/mL, MD = -85.8, 95%CI [-204.34;32.74], p = 0.16) [31], incidence of multiple organ dysfunction syndrome (17/220 vs 3/72, RR: 1.85, 95%CI [0.56;6.15], p = 0.31) [33], incidence of envenoming (31/35 vs 16/19, RR: 1.05, 95%CI [0.84;1.32], p = 0.66) [13], tissue necrosis (3/38 vs 1/19, RR: 0.75, 95%CI [0.14;4.12], p = 0.74) [13] and the occurrence of death or disability (14/53 vs 1/15, OR: 4.7, 95%CI [0.58;212], p = 0.16) [16].

Inconclusive evidence exists concerning the effects of tourniquet use on the duration of hospital stay. Madaki *et al*. found no significant difference in the duration of hospital stay between snakebite victims treated with a tourniquet and those receiving no first aid (6±2.6 days vs 6.3±3 days, MD = -0.3, 95%CI [-1.9;1.3], p = 0.71), while Michael *et al*. described a significant increase in the duration of hospital stay between snakebite victims treated with a tourniquet and those receiving no first aid (4.6±2.0 days vs 3.7±2.5 days, MD = 0.9, p = 0.04) [13,16]. Conflicting evidence was reported concerning the effects of tourniquet use on the amount of antivenom required. Amaral *et al*. reported no difference in the amount of antivenom required between snakebite victims treated with or without a tourniquet (139±56.4 mL vs 156.5±65.8 mL, MD = -17.5, 95%CI [-41.82;6.82], p = 0.16), while Madaki *et al*. found a significantly decreased amount of antivenom required in snakebite victims receiving a tourniquet (24.52±13.6 mL), compared to snakebite victims receiving no first aid (39.33±34.32 mL, MD = -14.81, p<0.01) and Michael *et al*. found a significantly increased amount of antivenom required in snakebite victims receiving a tourniquet compared to those receiving no tourniquet (20 [20;40] vs 20 [10;20], median[interquartile range (IQR)], p = 0.03) [13,16,22].

#### Incision of the bite wound

Bhat investigated the effects of incision of the bite wound, compared to no first aid treatment and found a significantly increased incidence of local swelling upon incision (13/13 vs 86/148, RR: 1.66, 95%CI [1.40;1.97], p<0.0001), but not of hemorrhagic syndrome (9/13 vs 98/148, RR: 1.05, 95%CI [0.71;1.53], p = 0.53) [30]. Furthermore, Michael *et al*. reported no difference between snakebite victims with incisions in the bite wound compared to victims receiving no first aid in the incidence of death or disability (2/8 vs 1/15, OR: 4.3, 95%CI [0.18;275], p = 0.53) or between snakebite victims with incisions in the bite wound compared to victims not receiving incisions in the bite wound for the amount of antivenom required (25.0 mL [0;35] vs 20.0 mL [20;35], median [IQR], p = 0.71) [16]. On the other hand, a statistically significant decrease in the duration of hospital stay in snakebite victims receiving incisions in the bite wound compared to not receiving incisions in the bite wound (2.9±1.6 days vs 4.6±2.2 days, MD = -1.70, p = 0.03) was demonstrated.

#### Snake stones

Madaki *et al*. could not show a significantly decreased incidence of envenoming in snakebite victims using snake stones (9/11) compared to those receiving no first aid (16/19, RR: 0.97, 95%CI [0.69;1.36], p = 0.87) [13]. Furthermore, a significantly decreased duration of hospital stay in snakebite victims treated with snake stones compared to those not receiving first aid (6.1±3.3 days vs 6.3±3 days, MD = -0.2, 95%CI [-2.57;2.17], p = 0.87) or to those not being treated with snake stones (2.5 vs 4, median, p = 0.09) could not be demonstrated [13,16]. Also a difference in the occurrence of death or disability between snakebite victims treated with snake stones or those receiving no first aid could not be demonstrated (2/4 vs 1/15, OR: 13, 95%CI [0.39;823], p = 0.11) [16].

In contrast, inconclusive results were reported for the amount of antivenom required. Madaki *et al*. reported a significantly decreased amount of antivenom required in snakebite victims treated with snake stones compared to those receiving no first aid (28.75±20.31 mL vs 39.33±34.32 mL, MD = -10.58, p<0.05), while Michael *et al*. reported no significant differences in the amount of antivenom required between snakebite victims treated with snake stones and those not treated with snake stones (30.0 [15;35] vs 20.0 [15;35], median[IQR], p = 0.71) [13,16].

#### Traditional medicine and concoctions

In the study of Madaki *et al*., the use of traditional medicine (both ingested or applied to the bite wound) in snakebite victims was not found to be significantly associated with a decreased occurrence of envenoming, compared to receiving no first aid (34/40 vs 16/19, RR: 1.01, 95%CI [0.8;1.28], p = 0.94) [13]. Furthermore, a decreased duration of hospital stay could not be demonstrated in snakebite victims receiving traditional medicine, compared to those receiving no first aid (6.9±2.6 days vs 6.3±3.0 days, MD = 0.6, 95%CI [-1.23;2.43], p = 0.52). This latter finding was confirmed by Michael *et al*., who observed no significant difference in the duration of hospital stay between snakebite victims treated with concoctions applied to the bite wound, compared to snakebite victims with no concoctions applied to the bite wound (5 vs 4, median, p = 0.6) or snakebite victims treated with concoctions ingested, compared to snakebite victims with no concoctions ingested (4 vs 4, median, p = 0.84) [16].

In contrast, a significantly increased odds for death or disability was shown in snakebite victims treated with concoctions applied to the bite wound (8/15), compared to snakebite victims with no concoctions applied to the bite wound (1/15, OR: 15, 95%CI [1.4;708], p = 0.01) and snakebite victims treated with concoctions ingested (6/10), compared to snakebite victims with no concoctions ingested (1/15, OR: 20, 95%CI [1.4;963], p = 0.009) [16].

Inconclusive reports were made concerning the use of traditional medicine and concoctions in snakebite victims on the amount of antivenom required. Madaki *et al*. reported a significantly decreased antivenom requirement in snakebite victims receiving traditional medicine, compared to those receiving no first aid (27.5±23.63 mL vs 39.33±34.32 mL, MD = -11.83, p<0.01), while Michael *et al*. found no significant difference in antivenom requirement between snakebite victims treated with applied concoctions, compared to those not treated with applied concoctions (30.0 [20;50] vs 20.0 [10;30], median[IQR], p = 0.07) or snakebite victims treated with ingested concoctions, compared to those not treated with ingested concoctions (30.0 [20;30] vs 20.0 [10;40], median[IQR], p = 0.13) [13,16].

#### Suction of the bite wound

The study by Michael *et al*. compared the effect of suction of a bite wound, and found no significant difference in the occurrence of death or disability, compared to no first aid (0/3 vs 1/15, RR: 1.33, 95%CI [0.07; 26.98], p = 0.85) [16]. Furthermore, a significant difference could not be demonstrated between snakebite victims treated by suction of the wound, compared to snakebite victims not treated by suction of the wound, concerning the amount of antivenom required (50 [0;60] vs 20 [20;30], median[IQR], p = 0.45) or the duration of hospital stay (6 vs 4, median, p = 0.7).

### Quality of evidence

An overview of the limitations in study design for the included studies individually, according to the GRADE approach is shown in Table 2. An overall assessment of the body of evidence is further elaborated below.

**Table 2:** Quality appraisal of the study designs of the included studies according to the GRADE approach.

**Experimental studies concerning the efficacy of pressure immobilization**					
**Author, Year**	**Lack of allocation concealment**	**Lack of blinding**	**Incomplete accounting of outcome events**	**Selective outcome reporting**	**Other limitations**
Anker, 1982 [23]	Unclear, no statement about allocation concealment	Highly likely lack of blinding due to the nature of the intervention	No, all participants were accounted for	No, the target outcomes were reported	Yes, unclear discrepancy in duration of treatments that might influence the maximal increase in radioactivity in the blood, and therefore also the outcome ‘time to reach 80% of the maximum radioactivity’
Anker, 1983 [24]	Unclear, no statement about allocation concealment	Highly likely lack of blinding due to the nature of the intervention	No, all participants were accounted for	No, the target outcomes were reported	Yes, unclear discrepancy in duration of treatments that might influence the outcome ‘% of max radioactivity in blood at release of treatment’, which has however not been extracted. All treatments lasted longer than 60 min, so the outcome % of maximum radioactivity in blood by 60 min should not be influenced
Howarth, 1994 [25]	Yes, partial within subjects design	Highly likely lack of blinding due to the nature of the intervention	No, all participants were accounted for	No, the target outcomes were reported	No
Tun Pe, 1994 [26]	Unclear, no statement about allocation concealment	Highly likely lack of blinding due to the nature of the intervention	No, all participants were accounted for	No, the target outcomes were reported	Yes, unclear discrepancy in duration of treatments that might influence the maximal increase in radioactivity in the blood, and therefore also the outcome ‘time to reach 80% of the maximum radioactivity’
**Experimental studies concerning the feasibility of pressure immobilization**					
**Author, Year**	**Lack of allocation concealment**	**Lack of blinding**	**Incomplete accounting of outcome events**	**Selective outcome reporting**	**Other limitations**
Canale, 2009 [27]	Yes, within subjects design	Highly likely lack of blinding due to the nature of the intervention, however, participants were blinded from pressure measurements	No, all participants were accounted for	No, the target outcomes were reported	Yes, performed in an artificial setting that lacks the stress of a real-life snakebite, study which recruited a majority of healthcare workers
Norris, 2005 [28]	No, laypeople were required not to have had first aid training of any kind before the study	No, the outcome measures were appropriate	No, all participants were accounted for	No, the target outcomes were reported	Yes, 20 volunteers were asked to each apply a bandage 5 times, and this was analyzed as n = 100 observations instead of n = 20 and the study was performed in an artificial setting, lacking the stress of a real-life snakebite
Simpson, 2008 [29]	Unclear, not stated how randomization took place	Yes, the nature of the experiments makes blinding highly unlikely, but this should not affect the outcome. Participants and assessors were blinded from pressure measurements during the experiment	No, all participants were accounted for	No, the target outcomes were reported	Yes, the study was performed in an artificial setting, lacking the stress of a real-life snakebite
**Observational studies concerning first aid measures applied in real-life snakebite**					
**Author, Year**	**Inappropriate eligibility criteria**	**Inappropriate methods for exposure and outcome variables**	**Not controlled for confounding**	**Incomplete or inadequate follow-up**	**Other limitations**
Amaral, 1998 [22]	Yes, all participants were Crotalus Durissus snakebite patients, but hospital-based sampling	No, snakebite was confirmed through snake identification or laboratory and clinical parameters	Yes, outcomes were analyzed with univariate tests	Yes, loss to follow up that was not accounted for in most of the outcomes.	Yes, retrospective study
Bhat, 1974 [30]	Yes, all participants were viper snakebite patients, but hospital-based sampling	No, snakebite was confirmed through snake identification or clinical parameters	Yes, outcomes were analyzed with univariate tests	No, all subjects were accounted for	No
França, 2003 [31]	Yes, all ‘mild’ and ‘moderate’ cases of envenoming were included, but hospital-based sampling	No, snakebite was confirmed through snake identification or clinical parameters	No, outcomes were analyzed with multivariate tests and corrected for age, gender, serum venom antigen concentration, site of bite and time interval for presentation	Yes, loss to follow up occurred without properly accounting for	No
Khin Ohn Lwin, 1984 [32]	Yes, all participants were Russell’s viper snakebite patients, but hospital-based sampling	No, Russell’s viper snakebite was confirmed with a snake venom ELISA	Yes, outcomes were analyzed with univariate tests	No, all subjects were accounted for	Yes, unclear from which location blood samples were taken
Madaki, 2005 [13]	Yes, all participants were snakebite patients, but hospital-based sampling	No, snakebite was verified through snake identification or clinical parameters	Yes, outcomes were analyzed with univariate tests	Unclear, as different combinations of first-aid measures were combined and compared, it is unclear whether all patients within a subgroup have been included in the analyses	Yes, retrospective study
Michael, 2011 [16]	Yes, all participants were snakebite patients, but hospital-based sampling	No, snakebite was verified through clinical parameters	Yes, the outcome death/disability was analyzed with a univariate test	Unclear, as different combinations of first-aid measures were combined and compared, it is unclear whether all patients within a subgroup have been included in the analyses	Yes, continuous and dichotomous data were analyzed differently: The outcome amount of antivenom requirement was compared between a first-aid treatment and no first-aid treatment/other first-aid treatments, while the outcome risk of death/disability was compared between a first-aid treatment and not receiving any first-aid treatment
Wang, 2014 [33]	Yes, all participants were Chinese cobra snakebite patients, but hospital-based sampling	No, snakebite was verified through snake identification and clinical parameters	Yes, outcomes were analyzed with univariate tests	No, all subjects were accounted for	Yes, data in table might not be correct for outcome MODS incidence (# cases and % cases are not the same), retrospective study

#### Experimental studies concerning the efficacy of pressure immobilization

The 4 studies assessing the efficacy of pressure immobilization were all experimental studies, resulting in a “high” initial quality level [23–26]. The quality of the evidence presented in the different studies was then appraised at the single study level for limitations in study design or execution, possibly leading to bias. The quality of the evidence was downgraded due to limitations in study design or execution (Table 2). Three of the 4 included studies made no statement about allocation concealment [23,24,26]. The study by Howarth *et al*. used a within subjects design, which is problematic for the intervention “rest vs walking”, as it is unclear whether the increase in tracer transit upon walking is actually due to the movement or might be due to other factors that appear over time, e.g. loosening of the bandage [25]. Furthermore, no statements were made about blinding in any of these studies, but the nature of the interventions makes blinding highly unlikely. In two studies, the major outcome studied, time to reach 80% of the maximal radioactivity in the blood, is likely to be influenced by the fact that the different first aid treatments were not applied for a fixed period of time [23,26].

Another unavoidable drawback of all of the included studies is the artificial setting of the experiments and the use of mock venoms. Therefore, the overall quality was downgraded for indirectness.

Finally, the overall quality was also downgraded for imprecision, due to limited sample sizes, low numbers of events and large variability of the results. There was no reason to downgrade for inconsistency or a risk of publication bias. The final level of quality for the experimental studies concerning the efficacy of pressure immobilization is “very low”, which means that any estimate of effect is very uncertain.

#### Experimental studies concerning the feasibility of pressure immobilization to be performed by laypeople

The 3 studies assessing the feasibility of pressure immobilization were all experimental studies, resulting in an initial “high” quality level [27–29]. The studies were then assessed at the single study level for limitations in study design or execution, and the quality of evidence was downgraded (Table 2). Canale *et al*. used a within subjects design [27], while Simpson *et al*. made no statement about how randomization took place [29]. None of the studies made a statement about blinding of the study design, but the nature of the interventions makes blinding highly unlikely. However, in two studies it was clearly stated that the test subjects were blinded from the pressure measurements [27,29]. In addition, the fact that all 3 studies took place in an artificial setting, lacking the stress of a real-life snakebite, is an unavoidable limitation of the studies. Canale *et al*. also used mostly healthcare workers, while first aid is mainly targeted at laypeople [27]. Finally, Norris *et al*. recruited 20 volunteers, but analyzed 5 repetitions by these volunteers as 100 distinct events, thus artificially increasing sample sizes and neglecting the possible effect of a learning curve [28].

Furthermore, the overall quality was also downgraded for imprecision, due to limited sample sizes, low numbers of events and large variability of the results. There was no reason to downgrade for indirectness, inconsistency or a risk of publication bias. The final level of quality for the experimental studies concerning the feasibility of pressure immobilization is “low”, which means that further research is very likely to have an important impact on our confidence in the estimate of effect and is likely to change the estimate.

#### Observational studies concerning first aid measures applied in real-life snakebite

The 7 studies included were observational studies, leading to an initial “low” quality level [13,16,22,30–33]. The quality of evidence was downgraded for limitations in study design or execution (Table 2). The included studies all used a hospital-based sampling strategy, thus possibly introducing selection bias, as the cases that do not make it to the hospital for various reasons are omitted from the analysis [13,16,22,30–33]. All but one study did not correct for confounding factors [13,16,22,30,32,33]. Furthermore, it was unclear for 2 studies whether all subjects were properly accounted for [13,16], while in 2 other studies, there was loss to follow up without properly accounting for [22,31]. In addition, Khin Ohn Lwin *et al*. did not report how blood samples were taken to measure serum venom levels [32]. In the study by Michael *et al*., different outcomes were compared to a different control group (i.e. subjects receiving no first aid or subjects not receiving a particular first aid treatment), without specifying why this is the case [16]. Finally, the data reported for incidence of multiple organ failure dysfunction syndrome by Wang *et al*. might be incorrect, as the amount of reported cases in the tourniquet and non-tourniquet group and the reported percentage of the total amount of snakebite victims are discrepant [33].

In addition, the overall quality of evidence was also downgraded because of imprecision due to limited sample sizes, low numbers of events and large variability of the results. There was no need to downgrade due to indirectness, inconsistency or a risk of publication bias. Therefore, the final level of quality for the observational studies concerning first aid measures is “very low”, which means that any estimate of effect is very uncertain.

## Discussion

This study aimed to summarize the best available evidence concerning the effectiveness and feasibility of first aid treatments for snakebites. A broad search strategy was used, to identify a wide range of first aid measures that are claimed to be effective. However, for several suggested first aid treatments, such as electroshock therapy or cryotherapy, no studies were found that met the predefined selection criteria. A total of 7 experimental and 7 observational studies that addressed the PICO question were identified.

The experimental studies all concern the pressure immobilization technique, based on the use of a crepe or elastic bandage. This technique received a lot of attention in Australia, and is being recommended in official Australian first aid guidelines [34,35]. However, the effectiveness of this technique has only been demonstrated in animal models [17,36], with evidence from human studies being limited to case reports [37,38]. Three of the studies on pressure immobilization efficacy meeting the selection criteria of this systematic review, favor a modified version of this technique, involving a localized cloth or rubber pad, firmly pressed on the site of the bite wound, with or without splinting, instead of a crepe or elastic bandage compressing the whole limb [23,24,26]. One study suggests that keeping a person still delays the spread of the venom [25]. However, the feasibility of correctly applying pressure immobilization using an elastic bandage is questionable, especially regarding the tension generated [27–29]. Intense training is warranted, but even then, retention is low [27,29]. Studies on the feasibility of applying a firmly strapped cloth or rubber pad are unavailable. Furthermore, it needs to be taken into account that the pressure immobilization technique might not be appropriate for any type of snake venom. The technique has a theoretical basis for limiting the spread of neurotoxic venoms, such as those produced by elapids, but less for necrotic venoms, such as those produced by vipers [39]. However, no controlled studies have been performed in real-life snakebite patients yet, leaving this controversy unresolved.

Studies concerning other first aid techniques were all observational. For tourniquets, most outcomes that were studied show no benefit of using a tourniquet in snakebite victims [13,16,22,30–33]. Moreover, the few outcomes that do show significant differences between tourniquet treated snakebite victims and victims receiving no tourniquet or no first aid show harmful effects of tourniquets on local symptoms [30,31,33]. For two outcomes, inconclusive evidence was found [13,16,22]. The bulk of evidence thus indicates that tourniquet use is not indicated for the treatment of snakebite.

The evidence available for other first aid measures is scarce, with evidence for the use of incisions, snake stones, traditional medicine, concoctions and suction being extracted from only 3 studies [13,16,30]. Concerning the use of incisions, 3 outcomes were not significantly different between snakebite victims treated with incisions and those treated without incisions or receiving no first aid [16,30]. One outcome, duration of hospital stay, differed in favor of incision, while another, local swelling, differed in favor of no first aid treatment. For snake stones, 3 outcomes did not differ between subjects treated with snake stones or those treated without snake stones or who received no first aid [13,16]. One other outcome, the amount of antivenom required, had inconclusive results. The use of traditional medicine or concoctions had no statistically significant effect for 2 outcomes, an inconclusive effect for 1 outcome, the amount of antivenom required, and a harmful effect for another outcome, the incidence of death or disability [13,16]. Finally, suction was shown to be ineffective for the treatment of snakebite on 3 outcomes [16]. In addition, there is a potential threat for the caregiver who could be exposed to the poison when performing oral suction. In conclusion, these alternative methods for the treatment of snakebite are most likely not beneficial and perhaps even harmful. Most of these management strategies are applied by traditional healers, who might be preferred over healthcare professionals in first instance. The use of this type of ineffective pre-hospital care might cause a delay in the presentation of the snakebite victim to healthcare facilities, further increasing the detrimental impact of the snakebite on morbidity and mortality. Habib *et al*. previously showed a 1% increase in odds of dying from snakebite for every 1 h delay in healthcare facility presentation in a case-control study of snakebite victims in north-eastern Nigeria [40]. Evidence concerning the time to application for specific first aid measures and their influence on the timing of presentation at a healthcare facility is currently unavailable.

This systematic review has some limitations. The fact that the best available evidence was collected for different first aid techniques led to the inclusion of studies with differing study designs, which implies substantial heterogeneity between studies. The study populations, interventions and outcomes assessed differed between studies, thus complicating the comparison between different first aid techniques. Therefore, it was both unfeasible and unwarranted to perform meta-analyses. Secondly, the sample sizes studied were small, limiting the generalizability of the conclusions made. Thirdly, substantial bias was present in the included studies, as discussed in the quality of evidence paragraph of the results section. Fourthly, the indirectness of the experimental studies on the efficacy of pressure immobilization, all performed using mock venoms, further limits our confidence in the reported results. Thus, the overall quality of the available evidence was low to very low, according to the GRADE approach [21]. Studies on the efficacy and feasibility of pressure immobilization in real-life snakebite victims are crucial to draw trustworthy conclusions concerning this technique.

The evidence collected in this systematic review has been used for the development of a first aid guideline for sub-Saharan Africa [41], according to the principles of Evidence-Based Practice [19], which is being updated in 2016. No new evidence, concerning first aid treatments for snake bites, could be identified in the 2016 update. This summary of best available evidence has been presented to a panel of first aid experts, who have made a recommendation, based on the available evidence, taking into account the needs and preferences of the target group, i.e. African laypeople encountering a case of snakebite. The only first aid measure that is supported with evidence is pressure immobilization, but it appeared difficult to apply this technique correctly. However, keeping a mock-bitten victim still had a beneficial effect on the spread of the venom [25]. In addition, the inhibition of venom spread by pressure immobilization might not be virtuous for venoms which induce localized necrosis and edema, such as those from e.g. vipers, which occur frequently in sub-Saharan Africa [1]. Unfortunately, no studies on the use of the pressure immobilization technique have been performed in sub-Saharan Africa yet. Therefore, the final recommendation is as follows: “Make sure no additional bites are encountered. Try to identify the snake, but do not try to catch it. Reassure the victim, tell him/her to lie down and move as little as possible. Contact the emergency services immediately. Remove any jewelry, watch or tight clothing. Immobilize the affected limb and control the victim’s vital parameters until the emergency services arrive.”

## Conclusion

This systematic review on first aid measures for the treatment of snakebite by lay first aid providers, has revealed that none of the in the literature suggested measures is proven to be both effective and feasible for the treatment of snakebite. The pressure immobilization technique was found to be effective but not feasible for laypeople. Therefore, evidence supporting a first aid guideline used in daily practice is limited to supportive therapy until professional help arrives. However, given the low quality of the evidence found, high quality studies concerning the efficacy and feasibility of different forms of pressure immobilization are warranted.

## Supporting Information

S1 Table PRISMA checklist. (PDF)

S1 File Search strategies. (DOCX)

S2 Table List of excluded studies and reason for exclusion. (PDF)

S3 Table Synthesis of findings. (PDF)
