# Supplementary material for: The Treatment of Snake Bites in a First Aid Setting: A Systematic Review
Source: PLoS Negl Trop Dis. 2016 Oct 17;10(10):e0005079. doi: 10.1371/journal.pntd.0005079 (PMC5066967; doi:10.1371/journal.pntd.0005079)
Supplement: S1 Table — (PDF) [file pntd.0005079.s001.pdf]

PRISMA 2009 Checklist

Section/topic	#	Checklist item	Reported on page #
**TITLE**			
Title	1	Identify the report as a systematic review, meta-analysis, or both.	p 1: “The treatment of snake bites in a first aid setting: A systematic review”
**ABSTRACT**			
Structured summary	2	Provide a structured summary including, as applicable: background; objectives; data sources; study eligibility criteria, participants, and interventions; study appraisal and synthesis methods; results; limitations; conclusions and implications of key findings; systematic review registration number.	p 2: Background: The worldwide burden of snakebite is high, especially in remote regions with lesser accessibility to professional healthcare. Therefore, adequate first aid for snakebite is of the outmost importance. A wide range of different first aid techniques have been suggested in literature, and are being used in practice. This systematic review aimed to summarize the best available evidence concerning effective and feasible first aid techniques for snakebite, which could then be used for the development of a first aid guideline used in daily practice, according to the principles of Evidence-Based Practice.<br>Methods: An independent, systematic literature screening by two authors in the Cochrane Library, MEDLINE and Embase resulted in 14 studies concerning first aid techniques for snakebite management, fulfilling our predefined selection criteria. Data was extracted and the body of evidence was appraised according to the GRADE approach.<br>Principal findings: The pressure immobilization technique was identified as the only evidence-based first aid technique with effectiveness on venom spread. However, additional studies suggest that proper application of this technique is not feasible for laypeople. Evidence concerning other suggested first aid measures, such as the application of a tourniquet, suggests avoiding the use of these techniques.<br>Conclusions: The practical recommendation for the treatment of snakebite in a first aid setting is to immobilize the victim, while awaiting the emergency services. However, given the low to very low quality of the data collected, large-scale randomized controlled trials concerning the efficacy and feasibility of different variations of the pressure immobilization technique are warranted.
**INTRODUCTION**			
Rationale	3	Describe the rationale for the review in the context of what is already known.	p 4: “In literature, many different techniques, and a combination thereof, are claimed to be effective for the treatment of snakebite”
Objectives	4	Provide an explicit statement of questions being addressed with reference to participants, interventions, comparisons, outcomes, and study design (PICOS).	p 5: For this, the following PICO question was formulated: In people with snakebites (P), is a certain first aid intervention (I), compared to another first aid intervention or no intervention (C), effective and feasible for laypeople as a first aid treatment to increase survival, tissue healing, functional recovery, pain, complications, time to resumption of usual activity, restoration to the pre-exposure condition, time to resolution of the symptoms or other health outcome measures (including adverse effects) (O)?
**METHODS**			
Protocol and registration	5	Indicate if a review protocol exists, if and where it can be accessed (e.g., Web address), and, if available, provide registration information including registration number.	p 5: “No protocol was filed prior to the preparation of the manuscript, however the methodology described in our previously published methodological charter was followed”

Eligibility criteria	6	Specify study characteristics (e.g., PICOS, length of follow-up) and report characteristics (e.g., years considered, language, publication status) used as criteria for eligibility, giving rationale.	p 6: "Following experimental study designs were included: (quasi or non-) randomized controlled trials, controlled before and after studies or controlled interrupted time series, if the data were available. For studies concerning the feasibility of first aid interventions, non-controlled before and after studies were also included, since this is typically measured with that type of study design. Observational studies of the following types were also included: cohort and case-control study, controlled before and after study or controlled interrupted time series, if the data were available. We excluded observational studies if the intervention was already studied in experimental studies, letters, comments, narrative reviews, case reports, cross-sectional studies, animal studies, ex vivo or in vitro studies, conference abstracts unless no other relevant data was available, studies reporting no quantitative data, studies reporting only means, but no standard deviations (SDs), effect sizes, p-values. Only studies reported in English were selected."
Information sources	7	Describe all information sources (e.g., databases with dates of coverage, contact with study authors to identify additional studies) in the search and date last searched.	p 5: "The following databases were searched for relevant studies from their date of inception to March 10, 2016: The Cochrane Library for clinical trials and systematic reviews, MEDLINE (using the PubMed interface) and Embase (via the Embase.com interface)". "The reference lists of included studies and also the first 20 similar articles in PubMed were screened for other relevant publications."
Search	8	Present full electronic search strategy for at least one database, including any limits used, such that it could be repeated.	S1 File
Study selection	9	State the process for selecting studies (i.e., screening, eligibility, included in systematic review, and, if applicable, included in the meta-analysis).	p 5: "The searches and study selection procedures were performed independently by two reviewers (BA and VB). Any discrepancy between the reviewers was resolved by consensus or by consulting a third reviewer (EDB)."
Data collection process	10	Describe method of data extraction from reports (e.g., piloted forms, independently, in duplicate) and any processes for obtaining and confirming data from investigators.	p 6: "Data concerning study design, study population, outcome measures (expressed as mean difference, odds ratio or risk ratio) and study quality were independently extracted from the included studies by two reviewers (BA and VB) using an in advance prepared form. Any discrepancy between the reviewers was resolved by consensus."
Data items	11	List and define all variables for which data were sought (e.g., PICOS, funding sources) and any assumptions and simplifications made.	p 6: "For the population (P), studies concerning people with snakebites or healthy volunteers with "mock" snakebites were included. The interventions (I) that were included in this systematic review were interventions for the first aid management of snakebites, that can be applied by laypeople without medical background. We excluded interventions for the management of snakebites that are not feasible to be performed in a first aid setting where laypeople are the first aid providers. We selected studies that compared (C) the interventions to any other first aid intervention or no intervention. Concerning the outcomes (O), we included (1) survival, functional recovery, pain, complications, time to resumption of usual activity, restoration of the pre-exposure condition, time to resolution of symptoms or other health outcome measures (including adverse effects) for studies involving snakebite victims, (2) spread of mock venom for studies investigating the efficacy of pressure immobilization and (3) quality of the bandage applied and tension generated for studies investigating the feasibility of pressure immobilization."

Risk of bias in individual studies	12	Describe methods used for assessing risk of bias of individual studies (including specification of whether this was done at the study or outcome level), and how this information is to be used in any data synthesis.	p 7: . "The evidence was then assessed for limitations in 5 domains, for which the quality of evidence could be downgraded, namely limitations in study design, indirectness, imprecision, inconsistency and reporting bias. Limitations in study design were assessed at the level of the individual study using the items listed by GRADE."
Summary measures	13	State the principal summary measures (e.g., risk ratio, difference in means).	p 7: "Data and p-values were extracted directly from the publications, unless it is stated that these were calculated from raw data available using the Review Manager software [20]. Outcomes from the selected studies without raw data or statement of significance were not extracted. Data are represented as mean±standard deviation (SD) or relative risk (RR) with 95% CI (confidence interval), unless otherwise stated."
Synthesis of results	14	Describe the methods of handling data and combining results of studies, if done, including measures of consistency (e.g., I^2) for each meta-analysis.	Not applicable
Risk of bias across studies	15	Specify any assessment of risk of bias that may affect the cumulative evidence (e.g., publication bias, selective reporting within studies).	p 7: ". The overall quality was assessed separately for (1) experimental studies concerning efficacy of pressure immobilization, (2) experimental studies concerning feasibility of the application of pressure immobilization to be performed by laypeople and (3) observational studies concerning other first aid techniques (tourniquet application, suction, traditional medicine, snake stones, incision of the bite wound)."
Additional analyses	16	Describe methods of additional analyses (e.g., sensitivity or subgroup analyses, meta-regression), if done, indicating which were pre-specified.	Not applicable
**RESULTS**			
Study selection	17	Give numbers of studies screened, assessed for eligibility, and included in the review, with reasons for exclusions at each stage, ideally with a flow diagram.	Fig 1 and p 7: "A search in The Cochrane Library, MEDLINE and Embase resulted in a total of 3893 retrieved references (Fig 1). After removing 956 (BA) and 1132 (VB) duplicates, the titles and abstracts of 2928 (BA) and 2761 (VB) records were screened on relevance regarding the PICO question. For 81 publications (BA) and 101 (VB) publications, a full-text was obtained and eligibility was assessed, resulting in 12 articles that matched the predefined selection criteria. The majority of publications excluded had an inappropriate study design. A search in the references and similar articles lists of these publications resulted in 2 additional publications matching the selection criteria, leading to a total of 14 included articles."
Study characteristics	18	For each study, present characteristics for which data were extracted (e.g., study size, PICOS, follow-up period) and provide the citations.	Table 1 and p 11: "Of the 14 included articles, 7 were experimental studies [23–29] and 7 were observational studies [13,16,22,30–33]. 4 experimental studies evaluated the efficacy of a first aid treatment, i.e. variants of the pressure immobilization technique, on simulated snake bites [23–26], while 3 others examined the feasibility of pressure immobilization to be performed by laypeople [27–29]. The observational studies all examined the outcomes of different applied first aid procedures in snakebite patients [13,16,22,30–33]. An overview of the study characteristics of the included studies is shown in Table 1"

Risk of bias within studies	19	Present data on risk of bias of each study and, if available, any outcome level assessment (see item 12).	Table 2 and p 17: "An overview of the limitations in study design for the included studies individually, according to the GRADE approach is shown in Table 2."
Results of individual studies	20	For all outcomes considered (benefits or harms), present, for each study: (a) simple summary data for each intervention group (b) effect estimates and confidence intervals, ideally with a forest plot.	S3 Table and p 13: "A structured synthesis of the findings from the included studies can be found in S3 Table, a narrative overview is given below."
Synthesis of results	21	Present results of each meta-analysis done, including confidence intervals and measures of consistency.	Not applicable
Risk of bias across studies	22	Present results of any assessment of risk of bias across studies (see Item 15).	p 22: "The final level of quality for the experimental studies concerning the efficacy of pressure immobilization is "very low", which means that any estimate of effect is very uncertain.", p 23: "The final level of quality for the experimental studies concerning the feasibility of pressure immobilization is "low", which means that further research is very likely to have an important impact on our confidence in the estimate of effect and is likely to change the estimate." and p23: "Therefore, the final level of quality for the observational studies concerning first aid measures is "very low", which means that any estimate of effect is very uncertain."
Additional analysis	23	Give results of additional analyses, if done (e.g., sensitivity or subgroup analyses, meta-regression [see Item 16]).	Not applicable
**DISCUSSION**			
Summary of evidence	24	Summarize the main findings including the strength of evidence for each main outcome; consider their relevance to key groups (e.g., healthcare providers, users, and policy makers).	p 25: "The evidence collected in this systematic review has been used for the development of a first aid guideline for sub-Saharan Africa [39], according to the principles of Evidence-Based Practice [19], which is being updated in 2016. No new evidence, concerning first aid treatments for snake bites, could be identified in the 2016 update. This summary of best available evidence has been presented to a panel of first aid experts, who have made a recommendation, based on the available evidence, taking into account the needs and preferences of the target group, i.e. African laypeople encountering a case of snakebite."
Limitations	25	Discuss limitations at study and outcome level (e.g., risk of bias), and at review-level (e.g., incomplete retrieval of identified research, reporting bias).	p23: "However, for several suggested first aid treatments, such as electroshock therapy or cryotherapy, no studies were found that met the predefined selection criteria." and p 24: "The evidence available for other first aid measures is scarce, with evidence for the use of incisions, snake stones, traditional medicine, concoctions and suction being extracted from only 3 studies." And p 25: "This systematic review has some limitations. The fact that the best available evidence was collected for different first aid techniques led to the inclusion of studies with differing study designs, which implies substantial heterogeneity between studies. The study populations, interventions and outcomes assessed differed between studies, thus complicating the comparison between different first aid techniques. Therefore, it was both unfeasible and unwarranted to perform meta-analyses. Secondly, the sample sizes studied were small, limiting the generalizability of the conclusions made. Thirdly, substantial bias was present in the included studies, as discussed in the quality of evidence paragraph of the results section. Fourthly, the indirectness of the experimental studies on the efficacy of pressure immobilization, all performed using mock venoms,

			further limits our confidence in the reported results. Thus, the overall quality of the available evidence was low to very low, according to the GRADE approach [21]. Studies on the efficacy and feasibility of pressure immobilization in real-life snakebite victims are crucial to draw trustworthy conclusions concerning this technique.”
Conclusions	26	Provide a general interpretation of the results in the context of other evidence, and implications for future research.	p 25: “This systematic review on first aid measures for the treatment of snakebite by lay first aid providers, has revealed that none of the in literature suggested measures is proven to be both effective and feasible for the treatment of snakebite. Therefore, evidence supporting a first aid guideline used in daily practice is limited to supportive therapy until professional help arrives. However, given the low quality of the evidence found, large scale studies concerning the efficacy and feasibility of different forms of pressure immobilization are warranted.”
**FUNDING**			
Funding	27	Describe sources of funding for the systematic review and other support (e.g., supply of data); role of funders for the systematic review.	Funding information was stated in the financial disclosure section of the submission system, as requested in the submission guidelines of the journal: “This work was made possible through funding from the Foundation for Scientific Research of the Belgian Red Cross-Flanders. All authors are employees at the Belgian Red Cross-Flanders and were involved in the development of this systematic review. The funders had no role in study design, data collection and analysis, decision to publish, or preparation of the manuscript.”

From: Moher D, Liberati A, Tetzlaff J, Altman DG, The PRISMA Group (2009). Preferred Reporting Items for Systematic Reviews and Meta-Analyses: The PRISMA Statement. PLoS Med 6(7): e1000097. doi:10.1371/journal.pmed1000097

For more information, visit: **www.prisma-statement.org**.
