# Supplementary material for: The Treatment of Snake Bites in a First Aid Setting: A Systematic Review
Source: PLoS Negl Trop Dis. 2016 Oct 17;10(10):e0005079. doi: 10.1371/journal.pntd.0005079 (PMC5066967; doi:10.1371/journal.pntd.0005079)
Supplement: S1 File — (DOCX) [file pntd.0005079.s002.docx]

### S1 File: Search strategies

**MEDLINE (via the PubMed interface):**

1. “Snake bites”[Mesh] OR “snake venoms”[Mesh] OR snakebite*[TIAB] OR snake bite*[TIAB] OR snake envenomation*[TIAB] OR snake venom*[TIAB]
2. "Compression bandages”[Mesh] OR bandage*[TIAB] OR pressure[Mesh] OR pressure*[TIAB] OR immobilization[Mesh] OR immobili*[TIAB] OR tourniquets[Mesh] OR tourniquet*[TIAB] OR suction[Mesh] OR irrigation[Mesh] OR extract*[TIAB] OR suction*[TIAB] OR suck*[TIAB] OR aspirat*[TIAB] OR irrigat*[TIAB] OR cryotherapy[Mesh] OR cryotherapy[TIAB] OR ice[TIAB] OR cold[TIAB] OR cut[TIAB] OR cutting[TIAB] OR splint*[TIAB] OR blackstone*[TIAB] OR snakestone*[TIAB] OR black stone*[TIAB] OR snake stone*[TIAB] OR “potassium permanganate”[MeSH] OR potassium permanganate[TIAB] OR “electroshock”[MeSH] OR electroshock*[TIAB] OR electri*[TIAB]
3. 1-2 AND

**Embase (via the Embase.com interface):**

1. Snakebite/exp ‘snake venom’/exp OR snakebite*:ab,ti OR (snake NEXT/1 bite*):ab,ti OR (snake NEXT/1 envenomation*):ab,ti OR (snake NEXT/1 venom*):ab,ti
2. ‘compression bandage’/exp OR bandage*:ab,ti OR pressure/exp OR pressure*:ab,ti OR immobilization/exp OR immobili*:ab,ti OR tourniquet/de OR tourniquet*:ab,ti OR suction/exp OR ‘wound irrigation’/exp OR extract*:ab,ti OR suction*:ab,ti OR suck*:ab,ti OR aspirat*:ab,ti OR irrigat*:ab,ti OR ice:ab,ti OR cryotherapy/exp OR cryotherapy:ab,ti OR cold:ab,ti OR cut:ab,ti OR cutting:ab,ti OR splint*:ab,ti OR blackstone*:ab,ti OR snakestone*:ab,ti OR (snake NEXT/1 stone*):ab,ti OR (black NEXT/1 stone*):ab,ti OR ‘permanganate potassium’/exp OR (potassium NEXT/1 permanganate):ab,ti OR ‘electric shock’/exp OR electroshock*:ab,ti OR electri*:ab,ti
3. 1-2 AND

**The Cochrane Library:**

[mh “snake bites”] OR [mh “snake venoms”] OR snakebite*:ti,ab,kw OR (snake NEXT bite*):ti,ab,kw OR (snake NEXT envenomation*):ti,ab,kw OR (snake NEXT venom*):ti,ab,kw
