# Supplementary material for: The Treatment of Snake Bites in a First Aid Setting: A Systematic Review
Source: PLoS Negl Trop Dis. 2016 Oct 17;10(10):e0005079. doi: 10.1371/journal.pntd.0005079 (PMC5066967; doi:10.1371/journal.pntd.0005079)
Supplement: S2 Table — (PDF) [file pntd.0005079.s003.pdf]

S2 Table: List of excluded studies and reason for exclusion

First author	Year	Title	Type of exclusion	Reason for exclusion
Adukauskiene	2011	Venomous snakebites	Design	Narrative review
Alberts	2004	Suction for venomous snake-bite: a study of 'mock venom' extraction in a human model	Intervention	No control group
American College of Medical Toxicology	2011	Pressure Immobilization After North American Crotalinae Snake Envenomation	Design	Narrative review/position statement
Anker	1982	First aid for snakebite	Design	Letter
Arnold	1975	Results of treatment of Crotalus envenomation	Design	Case reports
Arnold	1979	Controversies and hazards in the treatment of pit viper bites	Outcome	No comparison between treatments was made
Ashurst	2012	Approach and management of venomous snake bites: A guide for the primary care physician	Design	Guideline
Balmain	1982	Pantyhose compression band-age. First-aid measure for snake bite	Design	Case reports
Blackman	1992	Venomous snakebite: past, pre-sent, and future treatment options	Design	Narrative review
Bush	2000	Pressure immobilization delays mortality and increases intra-compartmental pressure after artificial intramuscular rattle-snake envenomation in a porcine model	Design	Animal study

Bush	2004	Effects of a negative pressure venom extraction device (Extractor) on local tissue injury after artificial rattlesnake envenomation in a porcine model	Design	Animal study
Bush	2004	Snakebite suction devices don't remove venom: they just suck	Design	Comment
Christensen	1969	The treatment of snakebite	Design	Narrative review
Clark	1971	Cryotherapy and corticosteroids in the treatment of rattlesnake bite	Design	Animal study
Cohen	1992	Local heat and cold application after eastern cottonmouth moccasin (Agkistrodon piscivorus) envenomation in the rat: effect on tissue injury.	Design	Animal study
Cox	2006	Concepts in crotaline snake envenomation management	Design	Narrative review
Currie	2006	Treatment of snakebite in Australia: the current evidence base and questions requiring collaborative multicentre prospective studies	Design	Narrative review
Currie	2008	Effectiveness of pressure-immobilization first aid for snakebite requires further study	Outcome	Outcome studied was how bandaging is performed, not effectiveness or feasibility of bandaging
Cutter	1940	Snake-Bites: A Compact Suction Kit	Intervention	No intervention studied

Davidson	2001	Sam splint for wrap and immobilization of snakebite	Design	Brief report
De	2000	Is torniquet use ineffective in the pre-hospital management of South American rattlesnake bite?	Design	Letter
De Haro	2012	Management of viper envenomation in Europe	Design	Conference abstract
Eatough	2008	Pressure immobilization continues to be underused in suspected snakebite in children	Design	Letter
Fix	1967	Venom extraction and yields from the North American coral snake, Micrurus fulvius	Intervention	Not concerning snakebite victims
Forks	1994	Evaluation and treatment of poisonous snakebites	Design	Narrative review
Frank	1971	Snakebite or frostbite: what are we doing? An evaluation of cryotherapy for envenomation	Design	Narrative review
German	2005	Pressure-immobilization bandages delay toxicity in a porcine model of eastern coral snake (Micrurus fulvius fulvius) envenomation	Design	Animal study
Gill	1968	Cryotherapy in the treatment of snake envenomation. VOL. 8, No. 9	Design	Animal study
Gill	1970	The evaluation of cryotherapy in the treatment of snake envenomization	Design	Animal study

Glass	1981	Cooling for first aid in snake bite	Design	Letter
Godpower	2012	The effect of pre-hospital care for venomous snakebite on out-come in Nigeria	Design	Conference abstract
Gold	1992	Venomous snakebites. Current concepts in diagnosis, treat-ment, and management	Design	Narrative review
Gold	1993	Snake venom extractors: a valu-able first aid tool	Design	Letter
Gold	1994	Snake venom poisoning in the United States: a review of ther-apeutic practice	Design	Narrative review
Gold	1999	Pressure immobilization for neurotoxic snake bites	Design	Letter
Gold	2004	North American snake enven-omation: Diagnosis, treatment, and management	Design	Narrative review
Gray	2003	Pressure immobilization of snakebite	Design	Letter
Grayson	1953	A technic for using suction in cases of snake bite	Design	Case reports
Heap	1991	The epidemiology of snake bite presenting to British Military Hospital Dharan during 1989	Design	Epidemiological study
Hodgson	1996	Biology and treatment of the mamba snakebite	Design	Case reports
Isbister	2006	Snake bite: A current approach to management	Design	Narrative review

Ismail	1983	Snake-bite--prevention and first-aid	Design	Guideline
Jenkins	1974	Physical therapy for snake venom poisoning	Intervention	No intervention studied
Jucker	1987	[Experiences in the treatment of bites by poisonous snakes with the 'Venomex', new suction and incision instrument]	Design	Case reports
Kleber	1998	[Adder bites in humans]	Language	German
Komesaroff	1983	First aid management of snake bite	Design	Letter
Kubisz	1974	Cold induced retraction of rep-tilase clots	Population	Not concerning snake-bite victims
Kubisz	1976	Platelet shape change induced by cold and the retraction of reptilase clots	Population	Not concerning snake-bite victims
Kuo	1972	Clinico-pathological studies on snakebites in Taiwan	Intervention	No intervention studied
Leopold	1960	Ineffectiveness of suction in re-moving snake venom from open wounds	Design	Animal study
Lockhart	1964	A newer method of treating snake bite	Design	Narrative review
Lockhart	1965	Break-through in snakebite	Design	Case reports
Lockhart	1965	Treatment of snakebite	Design	Narrative review/case reports
Macgregor	1963	September 1963: Snakebite	Design	Narrative review
Madsen	2010	Snake bites	Design	Narrative review
Martin	2003	Vipera berus snake bite and its management	Design	Narrative review

McLane	2007	Scientific and standardization committee communications: classification and nomenclature of disintegrins isolated from snake venoms	Population	Not concerning snake-bite victims
Meggs	2010	Pilot studies of pressure-immobilization bandages for rattlesnake envenomations	Design	Animal study
Meggs	2012	Snake bite first aid: Interest of experimental studies	Design	Conference abstract
Mion	2008	Cobra envenomation	Design	Narrative review
Mitra	1987	Snake bite in India and its management	Design	Narrative review
Mullins	1960	Successful management of cobra bite with cryotherapy	Design	Case reports
Munro	1978	Snake bite in children. A five year population study from south-east Queensland	Intervention	Epidemiological study
Murrell	1981	The effectiveness of the pressure/immobilization first aid technique in the case of a tiger snake bite	Design	Case reports
O'Connor	2011	Pressure immobilization bandages not indicated in the pre-hospital management of North American snakebites	Design	Letter
Pandey	2010	Impact of first aid training in management of snake bite victims in Madi valley	Design	Epidemiological study
Pantanowitza	1997	Tourniquets for snake bites	Design	Letter

Parrish	1955	Early excision and suction of snakebite wounds in dogs	Design	Animal study
Pearn	1981	First-aid for snake-bite: efficacy of a constrictive bandage with limb immobilization in the management of human envenomation	Design	Case reports
Pearn	1982	First aid in snake bite; comment on mock venom	Design	Letter
Pearn	1982	Pressure/immobilisation first aid treatment of snake bite	Design	Letter
Pope	1946	Treatment of poisoning with rattlesnake venom; experiments with negative pressure, tourniquet and bulb suction	Design	Animal study
Pozio	1988	First aid and subsequent treatment of adder bites	Language	Italian
Reid	1970	The principles of snakebite treatment	Design	Narrative review
Reitz	1986	Evaluation of the Venom Ex apparatus in the initial treatment of puff adder envenomation. A study in rabbits	Design	Animal study
Rogers	2002	Simulated field experience in the use of the Sam splint for pressure immobilization of snakebite	Design	Comment

Rogers	2005	Struan Sutherland's 'Rationali-sation of first-aid measures for elapid snakebite'--a commen-tary	Design	Comment
Rojnuckarin	2012	Diagnosis and management of venomous snakebites in South-east Asia	Design	Narrative review
Russell	1961	Incision and suction following injection of rattlesnake venom	Design	Animal study
Russell	1967	First-aid for snake venom poi-soning	Design	Guideline
Russell	1982	Pressure and immobilization for snakebite remains speculative	Design	Letter
Seifert	2011	Pressure bandaging for North American snake bite? No!	Design	Letter
Seifert	2011	Commentary: pressure bandag-ing for North American snake bite? No!	Design	Comment
Seifert	2012	Pressure bandaging with immo-bilization in crotalinae envenomation controversy	Design	Conference abstract
Seiler III	1994	Venomous snake bite: Current concepts of treatment	Design	Narrative review
Shulov	1969	The efficacy of suction as a method of treatment in snake-venom poisoning	Design	Animal study
Simpson	2007	The pediatric management of snakebite: The national proto-col	Design	Editorial

Sparger	1969	Problems in the management of rattlesnake bites	Design	Case reports
Stahnke	1966	Snake bite and cryotherapy	Design	Narrative review
Stewart	1981	First-aid treatment of poisonous snakebite: are currently recommended procedures justified?	Design	Narrative review
Stewart	2003	Snake bite in Australia: first aid and envenomation management	Design	Narrative review
Sutherland	1975	Treatment of snake bite in Australia. Some observations and recommendations	Design	Narrative review
Sutherland	1976	Treatment of snake bite in Australia and Papua New Guinea	Design	Narrative review
Sutherland	1979	Rationalisation of first-aid measures for elapid snakebite	Design	Animal study
Sutherland	1980	New first-aid measures for envenomation: with special reference to bites by the Sydney funnel-web spider (Atrax robustus)	Design	Narrative review
Sutherland	1980	Simple method to delay the movement from the site of injection of low molecular weight substances	Design	Letter
Sutherland	1981	First aid for cobra (Naja naja) bites	Design	Narrative review
Sutherland	1981	When do you remove first aid measures from an envenomed limb?	Design	Letter

Sutherland	1983	Prolonged use of pressure/immobilisation after snake bite	Design	Letter
Sutherland	1994	The pressure immobilisation technique	Design	Comment
Sutherland	1995	Pressure immobilisation for snakebite in southern Africa remains speculative	Design	Comment
Sutherland	2005	Rationalisation of first-aid measures for elapid snakebite. 1979	Design	Animal study
Tokish	2001	Crotalid envenomation: the southern Arizona experience	Outcome	No statistics performed
Tucker	1993	Compression bandaging for snake bite [4]	Design	Comment
Tun Pe	1987	The efficacy of tourniquets as a first-aid measure for Russell's viper bites in Burma	Intervention	No control group
Tun Pe	1995	Local compression pads as a first-aid measure for victims of bites by Russell's viper (Daboia russelii siamensis) in Myanmar	Intervention	No control group
Tun Pe	2000	Field trial of efficacy of local compression immobilization first-aid technique in Russell's viper (Daboia russelii siamensis) bite patients.	Intervention	No control group
Van den Enden	2003	Bites by venomous snakes	Design	Narrative review
Van Mierop	1976	Poisonous snakebite: a review. II. Symptomatology and treatment	Design	Narrative review

Wall	2012	British Military snake-bite guidelines: pressure immobilization	Design	Narrative review
Warrell	1993	Snake bite and snake venoms	Design	Editorial
Watt	1988	Tourniquet application after cobra bite: delay in the onset of neurotoxicity and the dangers of sudden release	Outcome	No quantitative expression of the outcome studied
Welch	2001	Collective review: Use of stun guns for venomous bites and stings: A review	Design	Narrative review
Wingert	1975	Diagnosis and management of envenomation by poisonous snakes	Design	Narrative review
Winkel	1999	Pressure immobilization for neurotoxic snake bites	Design	Letter
Winkel	2001	Strychnine, ammonia and gunpowder for snakebite - The end of an era	Design	Editorial
Yanamandra	2014	Traditional first aid in a case of snake bite: More harm than good	Design	Case reports
Zamudio	2000	Fang tip spread, puncture distance, and suction for snake bite	Intervention	Not concerning snakebite victims
Not available	1979	First aid for snake bite in Australia	Design	Comment/narrative review
