# Supplementary material for: The Treatment of Snake Bites in a First Aid Setting: A Systematic Review
Source: PLoS Negl Trop Dis. 2016 Oct 17;10(10):e0005079. doi: 10.1371/journal.pntd.0005079 (PMC5066967; doi:10.1371/journal.pntd.0005079)
Supplement: S3 Table — (PDF) [file pntd.0005079.s004.pdf]

S3 Table: Synthesis of findings

**Outcome**	**Comparison**	**Effect Size**	**#studies, # participants**	**Reference**
**Suction**				
Death or disability	Suction vs no first aid	Not statistically significant:<br>0/3 vs 1/15 §<br>RR: 1.33, 95%CI [0.07; 26.98] * ¥<br>(p=0.85)	1, 3 vs 15	Michael, 2011
Amount of anti-venom required (mL) (median [IQR])	Suction vs no suction	Not statistically significant:<br>50 [0;60] vs 20 [20;30] £<br>(p=0.45)	1, 3 vs 69 §	
Duration of hospital stay (days) (median)		Not statistically significant:<br>6 vs 4 £<br>(p=0.70)	1, 3 vs 69 §	
**Tourniquet**				
Local swelling	Tourniquet vs no first aid	Statistically significant:<br>78/78 vs 86/148 §<br>RR: 1.71, 95%CI, [1.49;1.96] *<br>(p<0.00001)<br>*If favor of no tourniquet*	1, 78 vs 148	Bhat, 1974
	Tourniquet with incision vs no first aid	Statistically significant:<br>71/71 vs 86/148 §<br>RR: 1.71, 95%CI, [1.49;1.96] *<br>(p<0.00001)<br>*In favor of no tourniquet*	1, 71 vs 148	
Hemorrhagic syndrome	Tourniquet vs no first aid	Not statistically significant:<br>49/78 vs 98/148 §<br>RR: 0.95, 95%CI, [0.77;1.17] *<br>(p=0.62)	1, 78 vs 148	
	Tourniquet with incision vs no first aid	Not statistically significant:<br>46/71 vs 98/148 §<br>RR: 0.98, 95%CI, [0.80;1.20] *<br>(p=0.84)	1, 71 vs 148	
Amount of anti-venom required (ml)	Tourniquet vs no tourniquet	Not statistically significant:<br>139±56.4 vs 156.5±65.8<br>MD: -17.5, 95%CI [-41.82;6.82] *<br>(p=0.16)	1, 45 vs 52 §	Amaral 1998
		Statistically significant:<br>20 [20;40] vs 20 [10;20] £ (median and IQR)<br>(p=0.03)<br>*In favor of no tourniquet use*	1, 53 vs 19 §	Michael, 2011
	Tourniquet vs no first aid	Statistically significant:<br>24.52±13.6 vs 39.33±34.32<br>MD: -14.81 ££<br>(p<0.01)<br>*In favor of tourniquet use*	1, 35 vs 19 §	Madaki, 2005
Acute renal failure	Tourniquet vs no tourniquet	Not statistically significant:<br>4/42 vs 4/52 §<br>RR: 1.24, 95%CI [0.33;4.66] * ¥<br>(p=0.75)	1, 42 vs 52	Amaral 1998
Acute respiratory failure		Not statistically significant:<br>3/35 vs 3/49 §<br>RR: 1.4, 95%CI [0.3;6.53] * ¥<br>(p=0.67)	1, 35 vs 49	
Death		Not statistically significant:<br>2/45 vs 3/52 §<br>RR: 0.77, 95%CI [0.13;4.41] * ¥	1, 45 vs 52	

		(p=0.77)		
Local edema		Not statistically significant:<br>17/42 vs 21/51 §<br>RR: 0.98, 95%CI [0.6;1.61] * ¥<br>(p=0.95)	1, 42 vs 51	
Envenoming	Tourniquet vs no first aid	Not statistically significant:<br>31/35 vs 16/19 §<br>RR: 1.05, 95%CI [0.84;1.32] * ¥<br>(p=0.66)	1, 35 vs 19 §	Madaki, 2005
Duration of hospital stay (days)		Not statistically significant:<br>6±2.6 vs 6.3±3<br>MD: -0.3, 95%CI [-1.9;1.3] * ¥<br>(p=0.71)		
		<u>Statistically significant:</u><br>4.6±2.0 vs 3.7±2.5<br>MD: 0.9 ££<br>(p=0.04)<br>*In favor of no tourniquet*	1, 53 vs 15 §	Michael, 2011
Tissue necrosis	Tourniquet/ Tourniquet with incisions vs no first aid	Not statistically significant:<br>3/38 vs 1/19 §<br>RR: 0.75, 95%CI [0.14;4.12] * ¥<br>(p=0.74)	1, 38 vs 19	Madaki, 2005
Death or disability	Tourniquet vs no tourniquet	Not statistically significant:<br>14/53 vs 1/15 §<br>OR: 4.7, 95%CI [0.58;212] ¥<br>(p=0.16)	1, 53 vs 15	Michael, 2011
Serum venom level before antivenom treatment (ng/ml)		Not statistically significant:<br>77.85±74.82 vs 60.88±39.39 λ ¤<br>MD: 16.97, 95%CI [-20.79;54.73] * ¥<br>(p=0.38)	1, 20 vs 17 §	Khin Ohn Lwin, 1984
		Not statistically significant:<br>197.7±230.4 vs 283.5±406.6<br>MD: -85.8, 95%CI [-204.34;32.74] *<br>(p=0.16)	1, 56 vs 61 §	França, 2003
Severity of local envenomation (mild vs moderate)		<u>Statistically significant</u>:<br>aOR: 4.31, 95%CI, [1.33;13.89]<br>(p=0.015) £££<br>*In favor of no tourniquet*		
Incidence of multiple organ dysfunction syndrome		Not statistically significant:<br>17/220 vs 3/72 §<br>RR: 1.85, 95%CI [0.56;6.15] * ¥<br>(p=0.31)	1, 220 vs 72	Wang, 2014
Skin grafting required		<u>Statistically significant</u>:<br>44/220 vs 7/72 §<br>RR: 2.06 ££<br>(p=0.046)<br>*In favor of no tourniquet*		
**Pressure immobilization**				
Efficacy				
Time to reach 80% maximal blood counts of radioactivity (min)	Elastic bandage and splinting vs no treatment	Not statistically significant:<br>26±17.06 vs 26±3.61 ¤<br>MD: 0.0, 95%CI [-19.73;19.73] * ¥<br>(p=1.00)	1, 3 vs 3 §	Anker, 1982
	Pressure cloth vs no treatment	<u>Statistically significant</u>:<br>74.3±3.79 vs 26±3.61 ¤<br>MD: 48.3, 95%CI [42.38;54.22] *<br>(p<0.00001)<br>*In favor of pressure cloth*		

	Treatment with rubber pressure pad and splinting vs no treatment	Statistically significant:<br>66.07±9.71 vs 42.38±5.01 ¤<br>MD: 23.69, 95%CI [17.53;29.85] *<br>(p<0.00001)<br>*In favor of rubber pressure pad and splinting*	1, 14 vs 8 §	Tun Pe, 1994
Amount of radioactivity in blood sample (% of maximal radioactivity in blood after 60 min)	Elastic bandage and splinting vs no treatment	Not statistically significant:<br>40.67±4.51 vs 46.33±16.17 ¤<br>MD: -5.66, 95%CI [-24.66;13.34] * ¥<br>(p=0.56)	1, 3 vs 3 §	Anker, 1983
	Pressure cloth vs no treatment	Statistically significant:<br>4.67±3.25 vs 46.33±16.17 ¤<br>MD: -41.66, 95%CI [-60.32;-23.0] *<br>(p<0.0001)<br>*In favor of pressure cloth*		
Transit of tracer	rest vs while walking	Lower limbs:<br>Statistically significant:<br>4/13 vs 9/9 §<br>RR: 0.34, 95%CI [0.16;0.73] *<br>(p=0.006)<br>*In favor of rest*	1, 13 vs 9 (within subjects)	Howarth, 1994
		Upper limbs:<br>Statistically significant:<br>7/13 vs 6/6 §<br>RR: 0.58, 95%CI [0.34;0.98] *<br>(p=0.04)<br>*In favor of rest*	1, 13 vs 6 (within subjects)	
Feasibility				
Tension generated within appropriate range	Training vs no training	Statistically significant:<br>18/36 vs 5/36 §<br>OR: 6.20 ££<br>(p=0.002)<br>*In favor of training*	1, 36 vs 36 (within subjects design)	Canale, 2009
Correct application of pressure immobilization technique	Lay people vs healthcare worker	Not statistically significant:<br>5/100 vs 13/100 §<br>RR: 0.38, 95%CI [0.14;1.04] * ¥<br>(p=0.06)	1, 100 vs 100	Norris, 2005
Correct bandage pressure applied		Not statistically significant:<br>14/100 vs 17/100 §<br>RR: 0.82, 95%CI [0.43; 1.58] * ¥<br>(p=0.56)		
Pressure applied with bandage (mmHg)	Intense training vs written instructions	Statistically significant:<br>57.7±17.0 vs 10.5±11.0<br>MD: 47.2, 95%CI [38.33;56.07] *<br>(p<0.00001)<br>*In favor of intense training*	1, 20 vs 20 §	Simpson, 2008
Tension within the correct range (55-70 mmHg)		Statistically significant:<br>12/20 vs 0/20 §<br>RR: 25.0, 95%CI [1.58;395.48] *<br>(p=0.02)<br>*In favor of intense training*		
	3 days after intense training vs 1h after intense training	Statistically significant:<br>5/20 (25%) vs 12/20 (60%) §<br>95%CI[6;44%]<br>(p<0.001)<br>*In favor of 1h after training*	1, 20 vs 20 (within subjects design)	
**Use of Concoctions/Traditional medicine**				
Envenoming	Traditional medicine (with or	Not statistically significant:<br>34/40 vs 16/19 §	1, 40 vs 19	Madaki, 2005

	without other first aid measures) vs no first aid	RR: 1.01, 95%CI [0.8;1.28] * ¥<br>(p=0.94)		
Amount of anti-venom required (mL)	Traditional medicine with tourniquet vs no first aid	Statistically significant:<br>27.5±23.63 vs 39.33±34.32<br>MD: -11.83 ££<br>(p<0.01)<br>*In favor of traditional medicine*	1, 17 vs 19 §	
	Concoction applied vs no concoction applied	Not statistically significant:<br>30.0 [20;50] vs 20.0 [10;30] £<br>(median [IQR])<br>(p=0.07)	1, 15 vs 57 §	Michael, 2011
	Concoction ingested vs no concoction ingested	Not statistically significant:<br>30.0 [20;30] vs 20.0 [10;40] £<br>(median [IQR])<br>(p=0.13)	1, 10 vs 62 §	
Duration of hospital stay (days)	Traditional medicine (with or without other first aid measures) vs no first aid	Not statistically significant:<br>6.9±2.6 vs 6.3±3.0<br>MD: 0.6, 95%CI [-1.23;2.43] *<br>(p=0.52)	1, 40 vs 19	Madaki, 2005
	Concoction applied vs no concoction	Not statistically significant:<br>5 vs 4 (median) £<br>(p=0.60)	1, 15 vs 57 §	Michael, 2011
	Concoction ingested vs no concoction ingested	Not statistically significant:<br>4 vs 4 (median) £<br>(p=0.84)	1, 10 vs 62 §	
Death/disability	Concoction applied vs no first aid	Statistically significant:<br>8/15 vs 1/15 §<br>OR: 15, 95%CI [1.4;708] ¥<br>(p=0.01)<br>*In favor of no first aid*	1, 15 vs 15	
	Concoction ingested vs no first aid	Statistically significant:<br>6/10 vs 1/15 §<br>OR: 20, 95%CI [1.4;963] ¥<br>(p=0.009)<br>*In favor of no first aid*	1, 10 vs 15	
**Snake stone**				
Envenoming	Snake stone vs no first aid	Not statistically significant:<br>9/11 vs 16/19 §<br>RR: 0.97, 95%CI [0.69;1.36] * ¥<br>(p=0.87)	1, 11 vs 19	Madaki, 2005
Amount of anti-venom required (mL)		Statistically significant:<br>28.75±20.31 vs 39.33±34.32<br>MD: -10.58 ££†<br>(p<0.05)<br>*In favor of snake stone*		
	Snake stone vs no snake stone	Not statistically significant:<br>30.0 [15;35] vs 20.0 [15;35] £<br>(median [IQR])<br>(p=0.71)	1, 4 vs 68 §	Michael, 2011
Duration of hospital stay (days)	Snake stone vs no first aid	Not statistically significant:<br>6.1±3.3 vs 6.3±3<br>MD: -0.2, 95%CI [-2.57;2.17] *<br>(p=0.87)	1, 11 vs 19	Madaki, 2005
	Snake stone vs no snake stone	Not statistically significant:<br>2.5 vs 4 (median) £<br>(p=0.09)	1, 4 vs 68 §	Michael, 2011

Death/disability	Snake stone vs no first aid	Not statistically significant:<br>2/4 vs 1/15 §<br>OR: 13, 95%CI [0.39;823]<br>(p=0.11)	1, 4 vs 15	
**Incision**				
Local swelling	Incision vs no first aid	Statistically significant:<br>13/13 vs 86/148 §<br>RR: 1.66, 95%CI, [1.40;1.97] *<br>(P<0.00001)<br>*With harm for incision*	1, 13 vs 148	Bhat, 1974
Hemorrhagic syndrome		Not statistically significant:<br>9/13 vs 98/148 §<br>RR: 1.05, 95%CI, [0.71;1.53] * ¥<br>(p=0.82)		
Death/disability		Not statistically significant:<br>2/8 vs 1/15 §<br>OR: 4.3, 95%CI [0.18;275] ¥<br>(p=0.53)	1, 8 vs 15	Michael, 2011
Amount of antivenom required (mL) (median [IQR])	Incision vs no incision	Not statistically significant:<br>25.0 [0;35] vs 20.0 [20;35] £<br>(p=0.71)	1, 8 vs 64 §	
Duration of hospital stay (days)		Statistically significant:<br>2.9±1.6 vs 4.6±2.2<br>MD: -1.70 (p=0.03) ££<br>*In favor of incision*		

MD = mean difference, SD = standard deviation, CI = confidence interval, IQR = interquartile range, RR = risk ratio, OR = odds ratio, aOR = adjusted odds ratio.

Mean ± SD (unless otherwise indicated)

* Calculations done by the reviewer(s) using Review Manager software

£ No effect size and CI available, imprecision due to variability of the results could not be verified

££ CI could not be calculated, imprecision due to variability of the results could not be verified

£££ No raw data available

¥ Imprecision (large variability of results)

§ Imprecision (limited sample size or low number of events)

λ data extracted from graph

¤ Mean and SD for each group calculated from subject data in Microsoft Excel
